# Asthma, Allergy and Eczema among Adults in Multifamily Houses in Stockholm (3-HE Study) - Associations with Building Characteristics, Home Environment and Energy Use for Heating

**DOI:** 10.1371/journal.pone.0112960

**Published:** 2014-12-05

**Authors:** Dan Norbäck, Erik Lampa, Karin Engvall

**Affiliations:** Department of Medical Science, Uppsala University, 75185 Uppsala, Sweden; Peking University, China

## Abstract

Risk factors for asthma, allergy and eczema were studied in a stratified random sample of adults in Stockholm. In 2005, 472 multifamily buildings (10,506 dwellings) were invited (one subject/dwelling) and 7,554 participated (73%). Associations were analyzed by multiple logistic regression, adjusting for gender, age, smoking, country of birth, income and years in the dwelling. In total, 11% had doctor's diagnosed asthma, 22% doctor's diagnosed allergy, 23% pollen allergy and 23% eczema. Doctor's diagnosed asthma was more common in dwellings with humid air (OR = 1.74) and mould odour (OR = 1.79). Doctor's diagnosed allergy was more common in buildings with supply exhaust air ventilation as compared to exhaust air only (OR = 1.45) and was associated with redecoration (OR = 1.48) and mould odour (OR = 2.35). Pollen allergy was less common in buildings using more energy for heating (OR = 0.75) and was associated with humid air (OR = 1.76) and mould odour (OR = 2.36). Eczema was more common in larger buildings (OR 1.07) and less common in buildings using more energy for heating (OR = 0.85) and was associated with water damage (OR = 1.47), humid air (OR = 1.73) and mould odour (OR = 2.01). Doctor's diagnosed allergy was less common in buildings with management accessibility both in the neighbourhood and in larger administrative divisions, as compared to management in the neighbourhood only (OR = 0.49; 95% CI 0.29–0.82). Pollen allergy was less common if the building maintenance was outsourced (OR = 0.67; 95% CI 0.51–0.88). Eczema was more common when management accessibility was only at the division level (OR = 1.49; 95% CI 1.06–2.11). In conclusions, asthma, allergy or eczema were more common in buildings using less energy for heating, in larger buildings and in dwellings with redecorations, mould odour, dampness and humid air. There is a need to reduce indoor chemical emissions and to control dampness. Energy saving may have consequences for allergy and eczema. More epidemiological studies are needed on building management organization.

## Introduction

Asthma and allergies have been on the rise in many countries over the past few decades and indoor exposure has been suggested as a possible cause. The dwelling is where we spend most of our time. Studies on indoor risk factors for asthma and allergies in the home environment among adults have focused mainly on building dampness and indoor mould [1] and environmental tobacco smoke (ETS) [2]–[4]. Another sources of indoor microbial contamination in homes, associated with adult asthma, includes storage of household waste for several days [5]. Chemical indoor factors at home associated with adult asthma include formaldehyde [2], [6], volatile organic compounds (VOC) [6] and emissions from indoor paint [7] and household cleaning sprays [8], leakage of gasoline emissions from garages [9], plastic wall materials and recent use of floor levelling plaster [10]. Moreover, use of air cleaners and air humidifiers at home has been associated with adult asthma [11]. Few studies are available on the association between ventilation flow in dwellings and asthma, but one study found a positive association between indoor levels of carbon dioxide (CO2), a proxy variable for ventilation flow, and asthmatic symptoms [6]. Another study found associations between wheeze and window pane condensation in winter, a proxy variable for high air humidity and low ventilation flow [12]. Other domestic risk factors for adult asthma include indoor allergens from cats, dogs, mice, house dust mites (HDM) and cockroaches [13]. Moreover, indoor fuel combustion is associated with asthma, especially in developing countries [4]. A few studies have investigated the associations between indoor factors at home and pollen allergy (hay fever) or furry pet allergy in adults. One found an association between dampness/indoor moulds at home and pollen allergy [14] and another found positive associations between recent indoor painting and furry pet allergy [12]. As for eczema and dermal symptoms in adults, domestic factors include dampness/indoor mould [14]–[17] and storage of organic household waste [5].

Over the past decade there has been growing concern regarding energy consumption in buildings in many countries, as a part of “Low Carbon Cities” programmes [18] striving for reduced greenhouse-gas emissions [19]. A recent review concluded that improvements in the physical fabric of the house, such as improved insulation, can lead to health improvements [20]. One intervention study showed that green renovation of dwellings could reduced energy consumption by 45% and improved the respiratory health in children [21]. Another intervention study found lower levels of asthma symptoms among asthmatic children living in homes that achieved better energy efficiency through retrofitted insulation and more effective heating [22]. Another study reported lower levels of house dust mite allergens in buildings designed for low energy use, as compared to normal buildings [23]. However, some studies indicate negative health effects of energy saving in dwellings. The potential health problems surrounding energy saving were addressed as early as in 1994 [24]. In the former East Germany, energy saving caused health disturbances due to new tight windows and doors [25] and increased concentrations of house dust mite allergens and mould spores [26]. One intervention study found that among asthmatic adults, some asthmatic symptoms were aggravated following energy retrofit work [27]. Another recent study concluded that tightening the building envelope in homes could lead to 20% more childhood asthma events, provided no adequate improvement of ventilation [28].

In Sweden, energy conservation issues were first addressed in the building code of 1975, and the energy use in residential buildings has been constant for many decades. The country's gross heated area increased by 40% from the 70s to 90s [29] after which it has increased further, indicating gradual increased energy efficiency for building heating. In 2010, the total annual energy consumption in Sweden was 395 TWh with residential buildings and commercial premises using 85 TWh for space heating and domestic water production. Of this, 42% was used in detached houses, 32% in multi-family buildings (flats) and 26% in commercial premises. In multifamily buildings, distance heating was the main source of building heating (91% of energy use); electric heating (4%) and oil combustion (1%) were rare. In detached houses, 37% used electric heating, 36% burnt wood/wood chips, 18% used distance heating and only 3% used oil. A full 46% of the detached houses have a heat pump installed. The hot water used for distance heating is produced mainly through the combustion in large units of renewable fuels such as wood chips (38%) garbage (19%) and peat (4%) in the cities and to a lesser extent by heat pumps (3%) [30]. Despite increased energy efficiency in Swedish buildings over past decades, we found no evaluation of the health consequences of this energy saving. Moreover, there are few large population studies exist where the associations between building characteristics, building management, indoor characteristics and energy consumption on the one hand and adult asthma and allergies on the other have been studied using mutual adjustment for various domestic environment factors, with additional adjustment for socioeconomic status.

The aim was to study associations between doctor's diagnosed asthma, doctor's diagnosed allergy, self-reported pollen allergy and self-reported eczema and selected building characteristics, building management routines and flat indoor characteristics. The hypothesis to be tested was whether the four health variables were associated with the following building data: building construction year, building size (no. of residents), type of ventilation, type of heat distribution, energy use for heating, type of ownership, type of maintenance management and management accessibility. Moreover, whether they were associated with the following flat/family data; crowdedness (no. of persons per room), family income, years living in the current dwelling, airing habits (airing index), recent redecoration, mouldy odour, water/dampness damage and indications of humid indoor air.

## Material and Methods

### Study design and study population

The study procedure and study protocol were approved by the Regional Ethical Review Board in Uppsala, Sweden. All participants gave their informed consent. An information letter sent together with the questionnaire stated that if the subjects answered and returned the questionnaire it meant they had given their informed consent.

In February-April 2005, 501 of a total of 14,465 (3.5%) multifamily buildings in Stockholm were selected from the central building register by stratified random sampling. Single-family houses (detached houses) and semi-detached houses were excluded, as well as 20 multifamily buildings used as nursing homes for the elderly. Data on building age and ownership was obtained from the building register. Strata were based on the construction year of the building classified into six classes (before 1961, 1961–75, 1976–84, 1985–1990, 1991–1997 and 1998–2003). The sampled buildings were selected from buildings with the registration code “exclusively multifamily buildings” which means that the buildings only contained flats. To obtain enough buildings in each class, the sampling proportion was lower for older buildings and higher for newer buildings. All dwellings in the selected 481 buildings were identified. The sampling proportions of selected flats were based on building size. In larger buildings, with more than 30 dwellings (N =  63), 30 dwellings were randomly selected into the study. In buildings up to 30 dwellings (N =  418) all dwellings were selected. In each selected dwelling, one randomly selected person (>18 years) who had lived in the flat for more than 6 months, was chosen by combining information in the building register with the civil registration register.

A self-administered postal questionnaire, the Stockholm Indoor Environment Questionnaire (SIEQ) [31], was sent to the selected subjects (N = 11,160 with the exception of 654 who did not actually live at the address. Responses came from 7,640 of 10,506 with correct addresses (73%). There were differences in response rate between the age classes of buildings, with the lowest response rate (66%) for buildings built in 1961–1975, and the highest (79%) for the newest buildings (1998–2003). Moreover, the response rate was lower in rented buildings (63%) as compared to buildings owned by the inhabitants (73%). A separate questionnaire was sent to building owners in April-May 2005, including questions about the building construction and maintenance organization. This information was received from 7,554 participants in dwellings in 472 buildings (98%).

### Assessment of building data

Data on building construction year and type of ownership was obtained from the building register. The questionnaire to the building owners contained questions on the building, including number of floors and number of flats, type of ventilation system and type of heating system. By combining the building register with the civil registration register, the number of residents in each building was obtained. Number of residents was divided by number of apartments to get the mean number of residents per square metre (persons/m2) for heated areas of the building. Heated area (m^2^, Atemp) was defined as areas with more than +10 °C room temperature in the building. In addition, the building owner questionnaire contained questions on management, operation and maintenance of the building. One question asked if management was by contract with the building owner or by an external company, or both. The other question was whether management was accessible for tenants at the division level or local level or both.

### Assessment of energy use for heating

In Sweden, it is compulsory for the building owners to provide information to the Government on energy consumption for their building. Information on the energy use for heating (kWh/m2, Atemp) was obtained mainly from this energy declaration form, available in the database *Gripen* at the National Board of Housing, Building and Planning [32]. Energy use for heating was calculated for heated areas of the building (m^2^, Atemp), defined as areas with more than +10 °C room temperature, and is the value used as the standard for energy declarations in Sweden. This roughly corresponds to the area inside the outer walls. Energy used for domestic hot water or household electricity is not included in energy use for heating. The energy declaration forms were mainly collected in 2008–2009. Measured data on electricity consumption and energy for heating for the buildings for March 2004-March 2005 was also gathered from the utility company Fortum Corporation. Data on energy uses for heating was controlled by certified energy consultants in the project group, recalculated if needed, and finally added into a new database. Special attention was given to make sure that energy measurements were for the individual building and not for the whole property, since a property can consist of several buildings. The methods for quality assurance of the energy data for these building has reported previously [33].

### Assessment of flat data

Information on the flat was obtained from the postal questionnaire, which included questions on size of the dwelling, number of rooms, environmental tobacco smoke at home (ETS), furry pet keeping, redecoration in the last 12 months, and water leakage, damage from damp, window pane condensation, slowly drying towels and airing habits (how much and how often windows were opened).

### Assessment of demographic and medical data

Information on medical symptoms and personal factors was obtained from the postal questionnaire, including age, gender, smoking habits, residency duration and airing and cleaning habits. There were four questions on asthma, allergy and eczema: Have you ever received a diagnosis of asthma from a medical doctor? Have you ever received an allergy diagnosis from a medical doctor? Have you ever had an allergy to pollen (hay fever)? Have you ever had eczema? Socioeconomic data such as income, citizenship, native country and year of immigration to Sweden, if not borne in Sweden, was obtained for each individual from Statistics, Sweden.

### Statistical methods

The number of independent variables that could be included in the statistical models was chosen according to the rule of thumb for a binary outcome [34]. To assess whether the set of included variables could be reduced we performed a hierarchical cluster analysis based on squared Spearman correlations as strongly correlated variables could create multicollinearity problems. Squared correlations were used since we were only interested in the magnitude of the correlations and not in whether they were positively or negatively correlated. From each cluster of variables we either created a summary score from the variables or chose one variable to represent the cluster. Window pane condensation and slow drying of towels in the bathroom were combined to one variable (1 = yes on any of the two variables, or else 0), and moisture damage and water damage during the past 5 years were combined into another variable (1 = yes on any of the two variables, or else 0).

Multiple logistic regression was used to relate the independent variables to the four dependent health variables. The standard errors were corrected for the clustering of individuals within buildings. One statistical model was used for each health variable, including all 14 selected building/flat variables, with additional adjustment for six potential confounders (gender, age, current smoker, passive smoking in the dwelling, annual income per family, foreign born yes/no), and number of years living in the current flat). One categorical variable was used combining active and passive smoking (coded zero if non-smoker with no ETS, coded 1 if non-smoker with ETS and coded 2 if current smoker). Continuous variables were modelled using restricted cubic splines with 3 knots. The non- linear terms were then either kept or removed based on a test of all non-linear terms equal to zero versus at least one non-linear term not equal to zero. Since there was some missing data on each independent variable, the main analysis was performed using multiple imputation. Imputations were done using the method of chained equations and 20 imputed data sets were created. Building variables were used in the imputation equations for the individual variables but not vice-versa. This was done to ensure that imputed building variables would be constant within buildings. The results were then compared with a complete case analysis. All analyses were made using R and STATA.

## Results

Among the participants, the proportion of females was 58%, the majority was 18–44 years old, and one-fifth was foreign-born and 15% were current smokers. In total, 11% had doctors' diagnosed asthma, 22% doctor's diagnosed allergy, 23% pollen allergy and 23% eczema (Table 1). Due to the stratified sample strategy, the number of buildings was almost equally distributed between the age classes in the stratified sample, with the highest proportion (25%) of the newest buildings (1998–2003). However, when weighing this against the total building stock in Stockholm, older buildings (built before 1976) were dominant and newer buildings (built after 1975) represented only 15% of all buildings in Stockholm (Table 2). In the sample, 52% of the buildings were owned by the inhabitant, 29% were publicly owned and 19% had a private landlord. Buildings owned by the inhabitants were more common in the newest buildings from 1998–2003, while publicly owned buildings were more common in buildings built in 1961–1975 and 1985–1990. Almost all buildings had district heating and were heated by water-borne radiators and the majority consumed 56–90 kWh/m2 and year for heating (Atemp). Nearly half of the building owners carried out their own management and a few had mixed work management. About half of the inhabitants had management accessibility in the neighbourhoods; a few had accessibility both in the neighbourhood and at the division level (Table 2). Data on family and flat level is reported in Table 3. The median number of persons per room was 0.67 (IQR range 0.5–1) and about half of the participants had lived more than five years in the current flat. In total, 30% had redecorated their flat during the past year, 7.8% reported damage from either moisture or water during the last five years and 9.2% reported humid air (either window condensation or long time to dry towels). Reports on mould odour were relatively uncommon (4.6%), and both pet keeping and environmental tobacco smoke at home (ETS) was relatively uncommon. Cats and dogs were the most common pets (Table 3).

**Table 1:** Personal characteristics of participants (n = 7554) in data and for Stockholm.

Characteristic		n	% in data set	% in Stockholm
Age	18–44	3452	45.8	48
	45– 64	2418	32.1	30
	> 64	1674	22.2	22
	Female gender	4401	58.3	57
	Foreign born	1695	22.5	21
	Current smoker	1082	14.7	18
Earned income by family/year a)	Low (<138 tkr)	1170	15.4	20
	Mean (138–485 tkr)	4526	60.1	63
	High (>485 tkr)	1832	24.3	17
Years lived in the dwelling	<than 1 year	82	1.1	2
	6–12 month	376	5.0	4
	1–3 years	1543	20.7	17
	4–5 years	1882	25.2	21
	6–10 years	1610	21.6	21
	more than 10 years	1972	26.4	36
Health variables	Doctor's diagnosed asthma	778	10.9	11
	Doctor's diagnosed allergy	1593	22.0	22
	Self-reported pollen allergy (hay fever)	1706	22.6	23
	Self-reported eczema	1615	22.7	23

a) Single households also count as family, tkr = thousands of Swedish krona (SEK).

**Table 2:** Building characteristics and management in the data set and in Stockholm area.

		Number of buildings (N = 472)			Number of participants (N = 7554)		
		n	%	Stockholm %	n	%	Stockholm %
Building construction year	−1960	65	13.8	74	955	12.7	68
	1961–1975	86	18.2	12	1318	17.5	17
	1976–1984	66	14.0	4	1113	14.8	5
	1985–1990	59	12.5	4	916	12.1	4
	1991–1997	80	17.0	5	1320	17.5	4
	1998–2003	116	24.6	2	1923	25.5	3
Number of flats	<29	115	33.5	55	2052	27.7	37
	30–59	112	24.2	22	1836	24.8	22
	60–99	86	18.6	11	1513	20.5	15
	≥100	110	23.8	13	1997	27.0	26
Type of ventilation	No mechanical ventilation	52	11.2	52	673	9.1	35
	Enhanced ventilation	23	5.0	7	339	4.6	11
	Exhaust air only	271	58.3	28	4475	60.5	38
	Supply/exhaust air	119	25.6	14	1913	25.9	16
Energy use for heating kWh/m^2^ (m^2^ A_Temp_) a)	Very low <55	22	5.9	2	419	6.7	3
	Low 56–90	201	53.7	34	3365	54.1	34
	Mean 91–124	116	31.0	43	1943	31.2	47
	High ≥125	35	9.4	21	498	8.0	16
Type of ownership	Public owned	137	29.0	29	2081	27.6	28
	Owned by inhabitant	244	51.7	32	4121	54.6	37
	Private landlord	91	19.3	39	1343	17.8	35
Maintenance management	By owners themselves	278	59.4	78	4193	56.2	76
	By contract	180	38.5	21	3116	41.7	22
	Mixed	10	2.1	2	157	2.1	1
Management accessibility	In the neighbourhood	222	49.0	55	3574	48.8	59
	On the division level	220	48.6	44	3561	48.6	40
	Both kinds of accessibility	11	2.4	1	187	2.6	2

a) m^2^ A_Temp_ means area with more than +10 C^0^.

**Table 3:** Number of participants (n = 7554), with respect to the flat and conditions under which participants live in the building, in data and in Stockholm.

Home characteristic		Number of flats (N = 7554)		
		n	%	Stockholm %
Persons per room a)	≤0, 5	2506	34.5	37
	>0,5–1	3792	52.2	51
	>1–1,5	659	9.1	8
	>1,5	308	4.2	5
Airing habits b)	Never/very little	923	13.1	11
	Little	2109	29.8	30
	Mean	834	11.8	14
	Very much	2085	29.5	31
	Extreme	1117	15.8	15
Decoration during last year	No redecoration	5267	70.3	68
	Yes, part of the flat	1663	22.1	23
	Yes, the whole flat	567	7.6	9
Keeping cats		671	9.0	9
Keeping dogs		385	5.2	5
Keeping rodents		157	2.1	1
Any type of furry pet		1147	15	14
Environmental tobacco smoke at home		321	5.9	7
Dampness over last 5 years in the dwelling c)		719	10.0	16
Water damage over last 5 years in the dwelling c)		541	7.8	11
Dampness or water leakage over last 5 years		1062	15.0	20
Window pane condensation in winter (yes, often) c)		274	3.8	7
Takes long time for towel/laundry to dry in the bathroom (yes, often) c)		494	6.7	12
Current humid air		663	9.2	15
Mouldy odour in the flat (yes, often) c)		351	4.6	7

a) Living room, bedroom and kitchen included.

b) Airing index based on how often and type of window opening.

c) Reported by inhabitant.

Associations between building/home environmental factors and the four health variables were analysed by weighted multiple logistic regression with imputation. One statistical model was used for each health variable, including all 15 selected building/flat variables, with additional adjustment for six potential confounders (gender, age, current smoker, annual income per family, foreign born yes/no, and number of years living in the current flat). When evaluating associations between the six confounders and four health variables, females hade more eczema (OR = 1.47; 95% CI 1.08–2.00; p = 0.02), doctor's diagnosed asthma (OR = 1.72; 95% CI 1.07–2.78; p = 0.03) and doctor's diagnosed allergy (OR = 1.77; 95% CI 1.20–2.60; p = 0.004). Younger subjects had more eczema (OR = 0.99; 95% CI 0.98–1.00 per year; p = 0.05) and hay fever (OR = 0.99; 95% CI 0.98–1.00 per year; p<0.001). There was a positive association between family income and eczema only (OR = 1.07; 96% CI 1.00–1.14 per 1000 SEK/Year; p = 0.04). Moreover, there was a tendency for foreign-born subjects to have less eczema (OR = 0.71; 95% CI 0.49–1.03; p = 0.07). There were no associations between number of years in the current dwelling and any of the four health variables.

Reports on humid air (p = 0.01), mouldy odour (p = 0.05) and type of management accessibility (p = 0.03) were significantly associated with doctor's diagnosed asthma, with a tendency of more asthma when management accessibility was only at the division level and less asthma if the accessibility was both in the neighbourhood and at the division level (Table 4). Building construction year, number of flats in the building, type of ventilation, energy use for space heating, type of ownership, type of maintenance management, environmental tobacco smoke (ETS), number of persons/room, airing habits, recent redecoration, damage from dampness or furry pet keeping were not associated with doctor's diagnosed asthma.

**Table 4:** Associations (p<0.2) between doctor's diagnosed allergy and independent variables (n = 7554).

Characteristic		OR(95%CI)	p-value
Type of ventilation			0.11
	No mechanical ventilation (S)	1.21(0.79–1.85)	
	Enhanced ventilation (FS)	1.07(0.60–1.89)	
	Exhaust air only (F)	1 (ref)	
	Supply/exhaust air only (FT)	1.44(1.07–1.94)	0.02
Management accessibility			0.09
	In the neighbourhood	1 (ref)	
	At the division level	1.08(0.74–1.57)	
	Both kinds of accessibility	0.51(0.26–0.99)	0.05
Room redecorated during the past year a)			0.02
	No, redecoration	1 (ref)	
	Yes, part of the flat	1.45(0.88–2.40)	
	Yes, the whole flat	1.50(1.11–2.05)	0.01
Mouldy odour in the flat		2.47(1.23–4.96)	0.01

Adjusted for gender, age, current smoker, foreign born, family income/year, number of years in current dwelling.

a) Reported by the inhabitant.

Participants living in buildings with both supply and exhaust air ventilation systems had more doctor's diagnosed allergy than those living in buildings with only exhaust air ventilation (p = 0.01). Moreover, subject living in recently decorated flats (p = 0.02) and those reporting mould odour at home (p = 0.02) had more doctor's diagnosed allergy while those who had management accessibility both in the neighbourhood and at the division level had less doctor's diagnosed allergy (p = 0.007) (Table 5). Building construction year, number of flats in the building, energy use for heating, type of ownership, type of maintenance management, number of rooms in the flat, ETS, number of persons/room, damage from damp, humid air or furry pet keeping were not associated with doctor's diagnosed allergy.

**Table 5:** Associations (p<0.2) between doctor's diagnosed asthma and independent variables (n = 7554).

Characteristic		OR(95%CI)	p-value
Management accessibility			0.07
	In the neighbourhood	1 (ref)	
	At the division level	1.39(0.95–2.05)	
	Both kinds of accessibility	0.34(0.07–1.70)	0.09
Current humid air		1.71(1.05–2.78)	0.03
Mouldy odour in the flat		1.71(0.94–3.09)	0.08

Adjusted for gender, age, current smoker, foreign born, family income/year, number of years in current dwelling.

Building construction years as a combined categorical variable was associated with pollen allergy (p = 0.04) with a tendency of less pollen allergy in building constructed 1961–1975. Buildings using more energy for space heating had less pollen allergy (p = 0.001) and those living in buildings with maintenance management by contract had less pollen allergy (p = 0.004) as compared to those whose owners took care of building maintenance themselves. Moreover, reports on humid air (p = 0.001) and mould odour (p = 0.001) were associated with pollen allergy while those living in flats with furry pets had less pollen allergy (p = 0.005) (Table 6). Number of flats, type of ventilation, type of ownership, type of management accessibility, and number of rooms in the flat, ETS, number of persons/room, airing habits, recent redecoration or damage from damp were not associated with pollen allergy.

**Table 6:** Associations (p<0.2) between self-reported pollen allergy (hay fever) and independent variables (n = 7554).

Characteristic		OR(95%CI)	p-value
Building construction year			0.06
	−1960	1.18(0.84–1.66)	
	1961–1975	0.78(0.57–1.08)	
	1976–1984	1.15(0.83–1.60)	
	1985–1990	1.02(0.75–1.37)	
	1991–1997	1.08(0.81–1.43)	
	1998–2004	1 (ref)	0.14
Energy use for heating (kWh/m^2^ and year)		0.76(0.63–0.92)	0.005
Maintenance management			0.02
	By owners themselves	1 (ref)	
	By contract	0.67(0.51–0.90)	
	Mixed	0.68(0.37–1.27)	0.008
Current humid air		1.74(1.24–2.44)	0.001
Mouldy odour in the flat		2.48(1.49–4.13)	0.001
Pet keeping (furry pets)		0.60(0.42–0.84)	0.003

Adjusted for gender, age, current smoker, foreign born, family income/year, number of years in current dwelling.

Subjects living in larger buildings (number of flats) had more eczema (p = 0.02) and those living in buildings using more energy for space heating had less eczema (p = 0.05). Moreover those with management accessibility at the division level had more eczema (p = 0.02) as compared to those with management accessibility in the neighbourhood. Finally, those reporting water damage during the five years (p = 0.05), humid air in the flat (p = 0.02) or mould odour (p = 0.03) had more eczema (Table 7). Building construction year, type of ventilation, type of ownership, type of maintenance management, ETS, number of persons per room, airing habits, recent redecoration and furry pet keeping were not associated with eczema.

**Table 7:** Associations (p<0.2) between self-reported eczema and independent variables (n = 7554).

Characteristic		OR(95%CI)	p-value
Number of flats in the building		1.07(1.00–1.14)	0.05
Energy use for heating (kWh/m^2^ and year)		0.86(0.72–1.00)	0.13
Management accessibility			0.12
	In the neighbourhood	1 (ref)	
	At the division level	1.42(1.01–2.00)	
	Both kinds of accessibility	1.26(0.78–2.03)	0.05
Water or humidity damage over last 5 years		1.57(1.05–2,33)	0.03
Current humid air		1.65(1.09–2.50)	0.02
Mouldy odour in the flat		1,96(1.05–3.68)	0.04

Adjusted for gender, age, current smoker, foreign born, family income/year, number of years in current dwelling.

## Discussion

Eleven of the fifteen selected building/flat variables - building construction year, number of flats, type of ventilation, energy use for heating, type of maintenance management, type of management accessibility, recent redecoration, damage from damp, humid air, mouldy odour and pet keeping - were either positively or negatively associated with asthma, allergy or eczema. Energy use for heating, type of maintenance management, type of management accessibility, humid air and mouldy odour were the most important exposure variables associated with more than one health variable. Most studies on health associations between indoor home environment and asthma, allergies and eczema have focused on environmental factors on the flat level. Few have studied factors on the building level and we found no previous studies on health associations between building management and management accessibility.

Epidemiological studies can be influenced by selection bias, recall bias (information bias) and type of statistical model. The participation rate was relatively high (74%). There were some differences in the proportion of participants between age classes of buildings, but they were relatively small. The study was based on a stratified random sample of the total population in Stockholm, with a higher sampling proportion for newer buildings and a lower sampling proportion for older buildings. The stratified random sampling influenced the prevalence of certain building related factors, such as the number of flats in the building, type of ventilation and type of ownership since these factors were associated with construction year or size of the building. However, mean age or proportion of females, foreign born, smokers, pet keepers or mean family income per year and mean time living in the current dwelling and the prevalence of asthma, allergies or eczema were similar in the stratified random sample as compared to the estimated total population in Stockholm. Thus, selection bias is unlikely to have had any major influence on the results from the study. Most data was collected from independent sources such as the population register (age, gender, foreign born, family income per year), the building register (construction year, number of flats, type of ownership) or a building questionnaire sent to the building owner (type of ventilation, type of maintenance management, type of heating system, type of management accessibility). Energy use for heating was determined independently by energy experts. However, variables concerning the flat were collected in the same questionnaire as the medical information and could be influenced by information bias. To reduce the number of statistical models, only four were used, one for each health variable. The advantage of this approach is that it reduces the risk for mass significance and the use of mutually adjusted models gives a better estimate of the true association, when adjusting for all exposure variables and potential confounders. Since the response rate was not 100% for each independent variable, imputation was applied. When comparing models with and without imputation, similar associations and estimates of the ORs were found (data not shown). In conclusion, it is less likely that the study was seriously hampered by selection bias, information bias or the selection of a particular statistical model.

The prevalence of doctor's diagnosed asthma was 10.9% in our study, performed in 2005. This is somewhat higher than the 9.3% doctor's diagnosed asthma reported from another population study in adults (20–79 years), performed in the Stockholm area in 2007 [35]. The prevalence of pollen allergy (hay fever) was 22.6% in our study, somewhat lower than the prevalence of adult allergic rhinitis (28%) in the Stockholm area 2006–2007 [36]. This latter study's definition of allergic rhinitis is similar to our definition of hay fever (pollen allergy). The prevalence of eczema in our study in adults (more than 18 y) was 22.7%, much lower than the prevalence of lifetime eczema in adults (16–75 y) (40.7%) reported from a population study in western Sweden [37]. Their question about eczema was somewhat different, since they asked about eczema or any kind of skin allergy, while we only asked about eczema.

The study confirms the conclusions from other studies suggesting that building dampness and moulds in homes are associated with asthma and to some extent allergies and eczema [1]. Water/dampness damage over the last five years was associated with eczema only, while indications of humid air was associated with doctor's diagnosed asthma, pollen allergy and eczema, and reports on mould odour showed associations with doctor's diagnosed allergy, hay fever and eczema. Humid air is an indicator of high relative air humidity in indoor air and can be influenced by low ventilation flow as well as the evaporation of water in flat. Mouldy odour at home has been reported to be consistently associated with onset of asthma [38] but we found only one previous study reporting an association between pollen allergy and mouldy odour [14]. The association in our study between dampness/moulds and eczema is in agreement with other population studies among adults in Sweden [14], [16], [17] and China [15]. One large multicenter study, mainly from Europe, found an association between adult eczema in the initial analysis, but the associations were not significant when adjusting for other indoor exposures and confounders [39].

New building material and indoor paints can emit formaldehyde and volatile organic compounds (VOC). The question about redecoration in our study revealed examples of redecoration, including change of floor materials, wall papers and indoor painting. Indoor painting is a common type of redecoration in dwellings [7]. We found an association between recent indoor redecoration and doctor's diagnosed allergy. This is in agreement with a previous study among Japanese female university students reporting an association between recent indoor painting and furry pet allergy [12].

Furry pet keeping was negatively associated with pollen allergy (hay fever). The most likely explanation is a selection effect, since subjects with allergies may avoid keeping furry pets. However, it has also been demonstrated that furry pet keeping may be associated with increased levels of endotoxin in house dust [40] and endotoxin may be protective against allergy, but the protective effect is mainly considered to occur in early childhood [41].

We found that subjects living in multifamily buildings using more energy for heating reported more pollen allergy and more eczema. In Sweden, energy saving in buildings has been undertaken for decades, with gradual reduced energy consumption per square meter of heated area. However, due to the global concern about greenhouse emissions, energy saving in buildings is still of major concern in both Sweden and abroad. Our data suggests that negative health consequences linked to energy saving in buildings should be considered. We found associations between energy consumption and pollen allergy and eczema after adjusting for a large number of confounder and other building/flat factors. Despite no information in our study on ventilation flow, only type of ventilation (which was adjusted for), it can be assumed that the buildings with higher energy consumption have better natural ventilation through air leakage in the building construction. In our study most multifamily building had a mechanical ventilation system but the majority only had exhaust ventilation. The potential of energy-saving is largest for a combined supply/exhaust ventilation system since heat exchangers can be used to transfer heat from the exhaust to the supply air ducts during the heating season. However, we found more pollen allergy in buildings with supply/exhaust ventilation, as compared to those with exhaust ventilation systems only. This illustrates a potential risk of supply/exhaust ventilation systems. The reason for the observed association is unclear, and should be confirmed in further studies. However it is important to avoid transport of pollen allergens and other outdoor particle pollutants through the supply air system to the indoor environment through sufficient air filtration and regular filter exchange.

We found that subjects in larger buildings reported more eczema. We found no other studies on associations between building size and adult eczema, but an association between degree of urbanisation and adult eczema has been reported in one Swedish population study from 2012 [37] as well as in a systematic review [42]. One explanation for our finding could be that areas with larger buildings have a larger degree of urbanization within the Stockholm area.

## Conclusions

Asthma, allergies or eczema among adults in Stockholm are more common in buildings with less use of energy for space heating, in larger buildings and in dwellings with redecoration, mould odour, dampness and humid air. There is still a need to reduce chemical emissions from new building materials and to control dampness and microbial growth in the dwellings. Increasing the energy efficiency in buildings may have consequences for health, and increased insulation of walls or roof should be combined with better air exchange, using an energy efficient mechanical ventilation system. More studies are needed on the role of building management for the inhabitants' asthma, allergies and eczema.
